# Hierarchical Bayesian Spatio–Temporal Analysis of Climatic and Socio–Economic Determinants of Rocky Mountain Spotted Fever

**DOI:** 10.1371/journal.pone.0150180

**Published:** 2016-03-04

**Authors:** Ram K. Raghavan, Douglas G. Goodin, Daniel Neises, Gary A. Anderson, Roman R. Ganta

**Affiliations:** 1 Kansas State Veterinary Diagnostic Laboratory, Department of Diagnostic Medicine and Pathobiology, Kansas State University, Manhattan, Kansas, United States of America; 2 Department of Geography, Kansas State University, Manhattan, Kansas, United States of America; 3 Bureau of Epidemiology and Public Health Informatics, Kansas Department of Health and Environment, Topeka, Kansas, United States of America; 4 Center for Excellence in Vector Borne Diseases, Department of Diagnostic Medicine and Pathobiology, Kansas State University, Manhattan, Kansas, United States of America; Cary Institute of Ecosystem Studies, UNITED STATES

## Abstract

This study aims to examine the spatio-temporal dynamics of Rocky Mountain spotted fever (RMSF) prevalence in four contiguous states of Midwestern United States, and to determine the impact of environmental and socio–economic factors associated with this disease. Bayesian hierarchical models were used to quantify space and time only trends and spatio–temporal interaction effect in the case reports submitted to the state health departments in the region. Various socio–economic, environmental and climatic covariates screened *a priori* in a bivariate procedure were added to a main–effects Bayesian model in progressive steps to evaluate important drivers of RMSF space-time patterns in the region. Our results show a steady increase in RMSF incidence over the study period to newer geographic areas, and the posterior probabilities of county-specific trends indicate clustering of high risk counties in the central and southern parts of the study region. At the spatial scale of a county, the prevalence levels of RMSF is influenced by poverty status, average relative humidity, and average land surface temperature (>35°C) in the region, and the relevance of these factors in the context of climate–change impacts on tick–borne diseases are discussed.

## 1. Introduction

Rocky Mountain spotted fever (RMSF) is a life–threatening fulminant tick-borne infection caused by the obligate intercellular bacterium *Rickettsia rickettsii* (Family: Rickettisiaceae, Order: Rickettsiales) [1]. This disease has been reported from most of the lower 48 states in the United States, with onsets typically occurring during the tick season (April through September) [1–3]. Despite the name, most RMSF cases occur in the lower Midwest and southern United States, with the states of Oklahoma, Arkansas, Missouri, Tennessee, and North and South Carolinas reporting most cases per year [4]. The disease is difficult to diagnose since symptoms do not develop until a few days well into infection [5], [6], and a lack of early treatment could lead to death [7]. Young children and the elderly are more frequently affected by RMSF [8], and although case fatality rates have declined during the last decade the incidences of RMSF has increased during the same time period in the United States [9].

Increased incidence of vector–borne diseases could be at least partly due to ongoing climate–change [10–12], and there is considerable interest in attributing climate–change effects on disease incidences. Arthropod life–cycles are highly regulated by climatic conditions, and the changes in recent climate patterns due to climate–change could affect incidences of tick-borne diseases in many ways. Some of these include changing spatial distribution of ticks and tick-borne pathogens [13], [14], and the persistence and replication rates of tick pathogens in their hosts [15]. Influential climatic factors behind increased incidence of RMSF are not clearly understood but are worth considering under a climate–change context.

At least three different tick species are confirmed vectors of RMSF in the United States [3], [16]. The population dynamics and spatial range of these ticks along with those of other arthropods may have changed in the past decade [17], [18] as a result of climate–change and other anthropogenic influences. The primary tick that transmits *R*. *rickettsii* to humans east of the Rocky Mountains is suggested to be the American dog tick, *Dermacentor variabilis* [3], although the role of *D*. *variabilis* ticks in the transmission has been contested since several recent reports have not detected the pathogen among field-collected ticks or those that were attached to humans or animals [19], [20]. The Rocky Mountain wood tick, *D*. *andersoni* is implicated in transmissions in the Rocky Mountain States and in the Pacific west. And, the brown dog tick, *Rhipicephalus sanguineus* has been shown to transmit this bacterium in the southwestern United States and along the United States–Mexico border [3], [21].

Determining the associations of influential climate and/or short–term weather factors with vector–borne diseases are problematic since they are often confounded with the effects of physical environment and socio–economic factors [10], [22]. A number of studies have shown relationship between various climatic (temperature, rainfall, humidity) factors and tick-borne illnesses but also physical environmental (land cover, landscape structure) and socio–economic factors [23], [24]. Therefore, attempts to determine climate associations with infectious diseases may benefit by simultaneously considering these additional factors. Also, abiotic determinants of vector–borne diseases are subject to changes due to influences of varying spatio–temporal factors, and incidences of vector–borne diseases have strong spatio–temporal dependencies, which necessitates evaluations of any associations to be conducted in a spatio–temporal context [25], [26]. Bayesian spatio–temporal models provide a flexible and robust platform for space-time analysis and also for evaluating covariate association with disease incidences between aggregated areal units such as a county. Such models have been used to study other tick-borne disease systems at a county scale [27], [28].

The purpose of this study was to evaluate the spatio–temporal pattern of RMSF incidence in the central United States spanning four contiguous states (Kansas, Missouri, Oklahoma and Arkansas) where this disease has noticeably increased over the past decade. Using case reports submitted to the respective state health departments over the years we asked the following specific questions; Is there an overall trend (increase, decrease or stable) for RMSF in the region comprising these four states? Are there areas within the region where there are the departures from the overall trend, or specifically high ‘risk-areas’? And, what are some of the influential factors–including any climate–change indices that are associated with RMSF incidence in this region?

## 2.0 Materials and Methods

### 2.1 Study area

The spatial extent considered in this study included the contiguous states of Kansas, Missouri, Oklahoma and Arkansas in central United States, which have recorded RMSF cases since the first identification of the disease, and has some of the highest number of incidences in the United States. Climate in the study area is transitional from east to west, and as well as south to north, with the southeastern region receiving progressively more rainfall than the west.

### 2.2 Data

#### 2.2.1 Ethics statement

The RMSF data used in this study was notifiable by individual state health departments to the Centers for Disease Control and Prevention (CDC), and were aggregated annually to their respective administrative units. No individually–identifiable information was collected for this study. The use of RMSF data was approved by the Internal Review Board at Kansas State University’s Office of Research Compliance (IRB #7465) and the study was considered exempt from the requirement for full review by the Missouri Department of Health and Senior Services (MDHSS) Institutional Review Board based on 45 CFR 46.101(b)(4).

#### 2.2.2 Epidemiological data

Annual county–level RMSF cases between years 2005 and 2014 were obtained from the state health departments of Kansas, Missouri, Arkansas and Oklahoma. Cases are classified into ‘confirmed’, ‘probable’, and ‘suspected’ categories per CDC guidelines. Case classification for RMSF changed once during the study period in 2008 and included the ‘suspected’ criteria for diagnosis. For the purposes of this study all three categories were considered to indicate positive RMSF diagnosis. In the case of Missouri, cases were predominantly aggregated at county–level; however, they also included three additional public health administrative units that were subsequently used in the disease mapping and Bayesian spatio–temporal analysis. Population data for these jurisdictions were provided by the MDHSS, and for all other jurisdictions an average value of county population recorded for years 2000 and 2010 by the US Census Bureau was used.

#### 2.2.3 Covariate data

Covariate data for this study was collected from three thematic groups; viz., physical environment, climate, and socio–economic status. For environmental data, the percentage land occupied by different cover types in each county was estimated in a geographical information systems (GIS) environment from the publicly available 2006 National Land Cover Dataset [29] (Table 1). Among climate variables, county averages of annual mean temperature; the maximum normalized vegetation index (NDVI); minimum land surface temperature (LST); mean LST; precipitation and relative humidity were extracted in a GIS environment for the corresponding years. The LST and NDVI estimates were derived from MODIS (Moderate Resolution Imaging Spectroradiometer) imagery [30]. Precipitation and relative humidity were derived from the Prediction of Worldwide Renewable Energy (POWER) web portal of the NASA Langley Research Center [31] (Table 2). Climate variables were extracted for a period roughly corresponding to the active tick–season in the region (May through August). For socioeconomic variables, the U.S. Census 2010 data on population and housing were obtained from the National Historical Geographic Information System (NHGIS), a publicly available online resource for U.S. Census Bureau’s historical and current population data [32] (Table 3). Identical census attribute information and geographic boundary files for counties were also obtained from the NHGIS. From the tables, 20 housing and 23 population related variables were extracted for each county by spatial query and joined to the census shapefiles using the common GIS codes.

**Table 1:** Physical environment variables screened in the study.

Source	Independent variables
NLCD (source: MRLC (2011); years^1^: 1992–2001; resolution^2^: 30 m; spatial scale^3^: 1:100,000)	Open water, developed—open space, developed—low intensity, developed—medium intensity, developed—high intensity, barren land, deciduous forest, evergreen forest, mixed forest, scrub/shrub, grassland/herbaceous, pasture/hay, cultivated crops, woody wetlands, emergent herbaceous wetland.

^1^ Years represent the time period during which satellite images of land cover were captured for creating the data set, including multiple images within a year.

^2^ Resolution indicates the fineness of ground data as captured by a satellite image, shorter resolution meaning higher clarity;

^3^ Spatial scale indicates the scale for which interpretations are appropriate.

**Table 2:** Climate variables evaluated in the study.

Source	Variable
NASA Moderate Resolution Imaging Spectroradiometer (MODIS)^¶^ Land Process Distributed Active Archive Center (LP DAAC)	Daytime land surface temperature (≥ 35°*C*, 28–34.9°*C*, 24.9–27.9°*C*, ≤ 25°*C*), Night time land surface temperature (≤ 16°*C*, 15.9–19.9°*C*, ≥ 20°*C*), Diurnal temperature range.
NASA Prediction of Worldwide Renewable Resources (POWER)	Normalized Difference Vegetation Index (NDVI), Daily maximum temperature, Daily minimum temperature, Daily average temperature, Dew point, Relative humidity, Diurnal temperature range.

^¶^ Several sixteen day composite MODIS images were downloaded for each year, for a period corresponding roughly to the tick season in North America (March–September), and county–level averages were estimated for different variables using pixels completely present within independent county boundaries.

**Table 3:** Population and housing variables evaluated in the study.

Census category	Independent variables^£^
Housing	*Housing units* (total housing units), *Tenure* (owner occupied, renter occupied), *Tenure (Historic or Latino Householder)* (owner occupied, renter occupied), *Race of householder* (white alone, Black or African American alone, Asian alone), *Household size* (1–person, 2–person, 3–person, 4–person, 5–person, 6–person, or 7–more person household), *Year structure built* (Built 2005 or later, 2000 to 2004, 1990 to 1999, 1980 or earlier^¶^). (20 variables).
Population	*Population* (total population), *Race* (White alone, Black or African American alone, Asian alone), *Household income in the past 12 months* (Less than $10,000, $10,000 to $14,999, and thirteen other variables representing $49,999 incremental income thereof up to $199,999, and $200,000 or more), *Poverty status in the previous 12 months* (income in the past 12 months below poverty level, income in the past 12 months at or above poverty level). (23 variables).

Definitions of different census variables can be found from their source (NHGIS) website at: https://www.nhgis.org/.

^£^ Observations for all the independent variables are counts, in continuous form, and recorded per areal unit (county). Items in italics are Census Table names, and items within parenthesis are independent variables evaluated in this study.

^¶^ The variable 1980 or earlier was derived by summing all the number of houses built prior to 1980 originally available in five–year increments in census.

### 2.3 Statistical analysis

#### 2.3.1 Covariate selection

Candidate explanatory variables to be included in the Bayesian hierarchical models were screened *a priori* in order to avoid model fitting issues. Several frequentist bivariate regression models were used to evaluate each variable independently and only variables that were significant at a liberal *p* ≤ 0.2 were kept for further analysis. A bivariate regression takes the form,

$$\left(Y_{ij}\right)=\beta_{0}+\;\beta_{k}vk_{ij}.$$

where *Y*_*ij*_ is RMSF relative risk, *β*_0_ the intercept coefficient, and *β*_*k*_ the coefficient for the explanatory variable *vk*_*ij*_ (*k* = **1**,…, *n*) and (*i* = 1, …, 105 *county*; *and j* = *year* 2005, …, 2014). Care was taken not to remove candidate variables that were deemed clinically relevant [33]. Among screened variables the presence of multicollinearity was tested by estimating the variance inflation factor (*VIF*) and all variables with a (*VIF* ≥ 10) were considered to indicate multicollinearity, in which case, one of the variables was dropped at a time until multicollinearity was absent. Non–linearity among independent variables was evaluated, and significant variables with non–linearity were categorized using cutoffs based on scatter–plots.

#### 2.3.2. Bayesian model specification

The observed number of RMSF cases in the study was notated as *Y*_*ij*_ among *N*_*ij*_ individuals at risk in the population of county *i*, diagnosed with RMSF in year *j*. *Y*_*ij*_ was assumed to follow a Poisson approximation, (*Y*_*ij*_) ~ *Poisson* (*E*_*ij*_ *θ*_*ij*_), where *E*_*ij*_ is the expected number of the population at risk for RMSF and *θ*_*ij*_ is the relative risk. Although a Binomial approximation could have been used in this context since *n* is known, relatively only few RMSF infections occur randomly over time with different age groups, whose members vary throughout the study period due to aging; therefore, Poisson distribution was chosen over Binomial since the latter assumes constant population of individuals. And, since RMSF prevalence is disproportionate among different age groups [4], standardized rates were calculated assuming five age classes *l*, (< 5, 5–19, 20–45, 46–65 and > 65). Mapping crude rates can be non–informative or misleading when population in some areal units are small, resulting in large posterior estimates, which in turn render it difficult to distinguish chance variability from genuine differences. The expected number of RMSF cases was therefore calculated by

$$E_{ij}=\sum_{l}^{}n_{ijl}\frac{\sum_{i}\sum_{ijl}Y_{ijl}}{\sum_{i}\sum_{ijl}N_{ijl}}.$$

A logit link function in an extended generalized linear model (GLM) structure was used that incorporated stochastic spatial and temporal functions and as well as different covariate effects. We first fitted a parametric spatio–temporal model with various random terms to act as surrogate indicators of unobserved risk factors that vary over time, space or both. In a second model, the parametric terms were replaced with nonparametric terms to assess any model improvements. In a third step, we extended the second model with different covariates to explain spatio-temporal patterns.

The parametric model was notated as following,

$$Log\left(\theta_{ij}\right)=\alpha +u_{i}+v_{i}+\gamma_{j}+\Psi_{ij}.$$

Where, *α* (intercept term) represents the mean prevalence of RMSF in all counties in all years, and *u*_*i*_ and *v*_*i*_ are random terms accounting for spatially structured variation in RMSF prevalence and unstructured heterogeneity, respectively. No interaction was assumed to exist between *u*_*i*_ and *v*_*i*_, and these terms were assigned *u*_*i*_ ~ *CAR*, and $v_{i}\sim Normal\;\left(0,\;\sigma_{v}^{2}\right)$ priors. Spatial dependence in *u*_*i*_ was applied by assuming a conditional autoregressive model (*CAR*)(*γ*) with a Gaussian distribution, which implies that each *u*_*i*_ is conditional on the neighbor *u*_*j*_ with variance $\left(\sigma_{i}^{2}\right)$ dependent on the number of neighboring counties *n*_*i*_ of county *i*, i.e.,

$$u_{i}|u,\;j\;neighbor\;of\;i\sim N\left[\frac{1}{n_{i}}\gamma \sum_{j=1}^{n_{i}}u_{j},\frac{\sigma_{i}^{2}}{n_{i}}\right].$$

The γ_*j*_ term measured the purely temporal and a linear time trend in the data, which assumed no temporal structure *a priori*. An independent mean–zero normal prior with unknown variance $\sigma_{\gamma }^{2}$ was used for *γ*. In the same model, in order to account for spatio–temporal interaction effects in RMSF prevalence, a spatially structured *Ψ*_*ij*_ term modeled as an intrinsic Gaussian Markov random field (IGMRF) [34] was included, with a joint prior density for *ψ* = (*ψ*_1_,…,*ψ*_*I*_) written as

$$\pi \left(\psi |\sigma_{\psi }^{2}\right)\;\propto \;\exp\left[-\frac{1}{2\sigma_{\psi }^{2}}\sum_{i\sim i^{\prime }}{\left(\psi_{i}-\psi_{i^{\prime }}\right)}^{2}\right].$$

A major limitation with the above described model is the assumption that there is a linear time trend in each region. Therefore, in a second model the linearity assumption was dropped and a nonparametric term, *β*_*j*_ was included, whose prior density can be written as

$$\pi \left(\beta |\sigma_{\beta }^{2}\right)\;\propto \;\exp\left[-\frac{1}{2\sigma_{\beta }^{2}}\sum_{t=2}^{T}{\left(\beta_{t}-\beta_{t-1}\right)}^{2}\right].$$

The *β* term quantified the overall time trend for the region. In addition, a nonparametric Type–IV interaction prior [25], [35] was assigned to the *Ψ*_*ij*_ term, which is notated as

$$\pi \left(\psi |\sigma_{\psi }^{2}\right)\;\propto \;\exp\left[-\frac{1}{2\sigma_{\psi }^{2}}\sum_{t=3}^{T}\sum_{i\sim i^{\prime }}^{I}{\{\left(\psi_{it}-2\psi_{i,t-1}+\psi_{i^{},t-2}\right)-\left(\psi_{i^{\prime },t-2}-2\psi_{i^{\prime },t-1}+\psi_{i^{\prime },t-2}\right)\}}^{2}\right].$$

For the extended models with covariate terms, different covariates were included to the second model in several steps, starting with a model that included all covariates screened in the bivariate procedure that retained significance at a liberal (*p* ≤ 0.2) value followed by the removal of one variable at each step. Individual covariates were retained in the model unless their removal resulted in the increase of Deviance Information Criterion *(DIC)* value by 5 units or more. The removed covariates did not re–enter the model and all covariates were assigned noninformative priors, $Normal\;\left(0,\;\sigma_{v}^{2}\right)$.

The spatiotemporal interaction term, *Ψ*_*ij*_ in the covariate model measured the trend after accounting for purely spatial (*α* + *u*_*i*_ + *v*_*i*_) and purely temporal effects (*β*_*ij*_) in addition to the covariate effects, and reveals the variation in RMSF incidence trends across counties over time. The differential trend (the difference between overall trend and local trend) of a county *i* for a given year *j* can also be estimated from *Ψ*_*ij*_, with *Ψ*_*ij*_ > 0 indicating steeper than overall trend and *Ψ*_*ij*_ < 0 indicating a less steeper the overall trend. Counties where *Ψ*_*ij*_ = 0 are areas where the trends are equal [36].

Model selection criteria in this study used two criteria, the *DIC* value and the logarithmic score *(LS)*. The former is the tool of Bayesian model choice for selecting the most parsimonious model after penalizing for model complexity [37], however *DIC* can be problematic in models that consider many random effects [38]. Thus, in addition to *DIC* the *LS* of each model was computed to assess predictive quality of models [39], [40], which is represented by *LS* = −*log*(*π*_*ij*_), where *π*_*ij*_ = *pr*(*Y*_*ij*_ = *y*_*ij*_ | *y*_-ij_) denotes the cross-validated predictive probability. A smaller *LS* indicates better predictive quality of a model.

All model posterior parameters were estimated using a Bayesian framework implemented using R–INLA software [41], and the median estimates from the posterior distribution and their corresponding uncertainty measures [95% Credible Intervals (CrI)] were recorded.

## 3. Results

**Fig 1:**
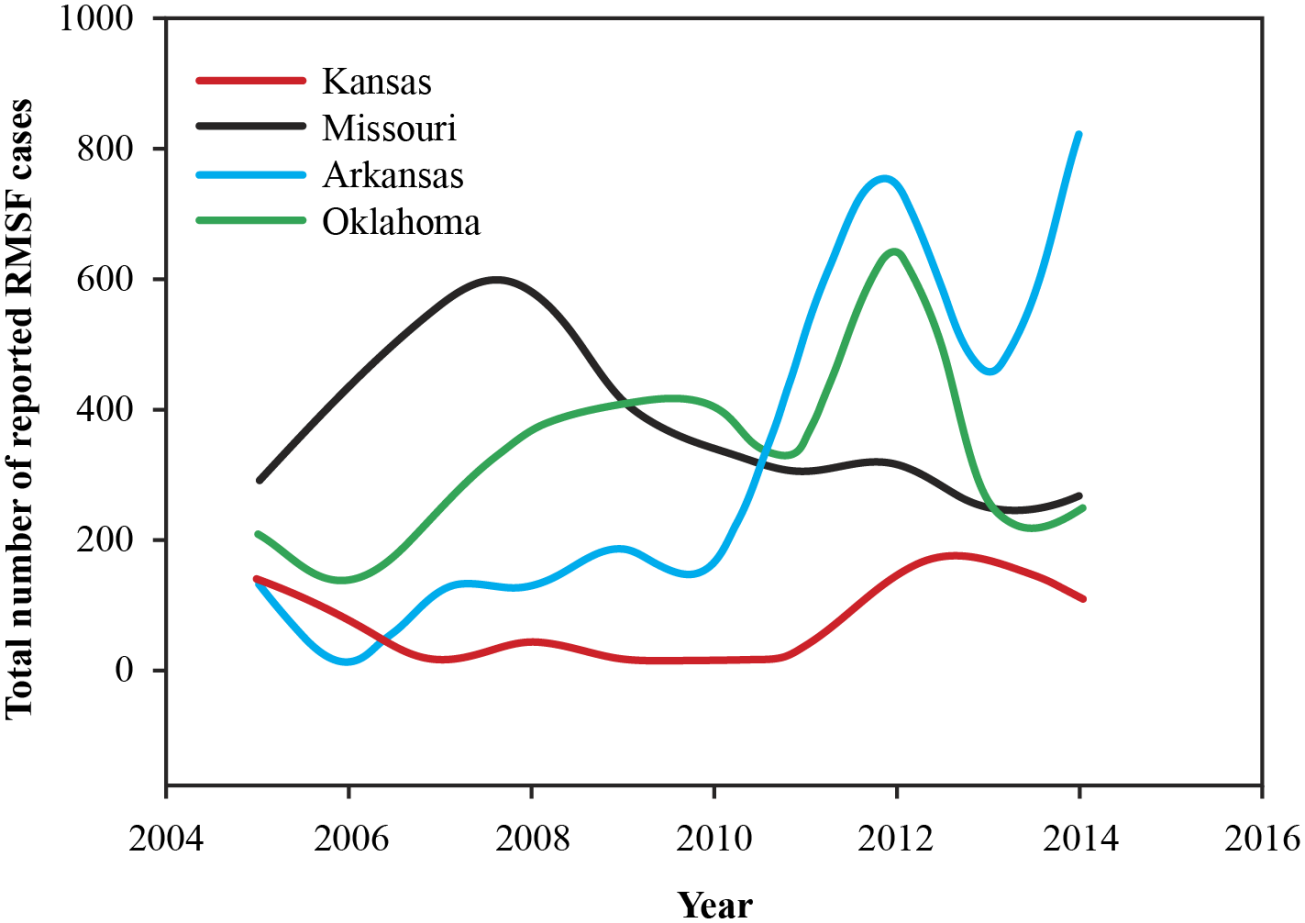
Plot of reported number of cases submitted to different state health departments in the study region.

From January 1, 2005 to December 31, 2014, there were a total of (*n* = 11,062) confirmed, probable and suspected cases of RMSF reported to the health departments of the four states included in the study. Missouri recorded the most number of cases (*n* = 3,766) followed by Arkansas (*n* = 3,271) and Oklahoma (*n* = 3,271), and Kansas recorded the least number of cases (*n* = 754) among the four states during this period. Throughout the study period, and in all four states males were diagnosed at a higher rate than females, with a male female ratio averaging 2.2:1 for all states. Also, the disease was more common among the 46–64 year old age group followed by the ≥65 year old age group. A plot of case numbers in the four states during the study period is illustrated in Fig 1. A general upward trend for cases can be observed for Arkansas and Oklahoma, while in Missouri the case numbers have fallen since 2007–2008 period with only a small increase in 2014, and Kansas is largely constant throughout the study period. Also, with the exception of Missouri, all three states have recorded a marked increase in the number of cases during the year 2012. Missouri experienced a similar peak in the year 2007.

Of all the variables evaluated in the bivariate screening procedure in this study, five retained significance at a liberal *p* ≤ 0.2 level (Table 4). Two variables, “Year structure built in 2005 or later” and “Year structure built in 2000–2005” were revealed as collinear among the selected variables. Non–linearity was not observed among the selected variables. Numerical results of Bayesian hierarchical models, the posterior Bayes median estimates and their corresponding 95% CrI are present in Table 5. From Table 6, we see that Model– 1 has the highest deviance information criterion (DIC) value pointing to the presence of additional space–time structure to the data beyond just purely spatial and temporal main effects. The addition of nonparametric terms to the fixed effect spatio–temporal model markedly reduced the *DIC* values, and the subsequent addition of covariate terms further improved the spatio–temporal model performance. ‘Income in the past 12 months below poverty level’ (henceforth, poverty–status), ‘average relative humidity’ and ‘average daytime land surface temperature ≥ 35°C’ were retained in the final, best fitting Bayesian hierarchical covariate model. This showed that the covariate terms are competing to explain the space–time structure in the dataset that are not captured by the main effects alone. Even though the covariate model is not the most parsimonious it was the best among all the models considered, and all interpretations were made based on this model alone.

**Table 4:** Results of bivariate regression analysis and candidate variables (*p* ≤ 0.2).

Covariate	Estimate	S.E	*p–value*
Income in the past 12 months below poverty level	0.89	0.31	0.01
Relative humidity	0.62	0.12	0.03
*Daytime land surface temperature*			
≥ 35°*C*,	–0.61	–0.13	0.03
28 − 34.9°*C*	–1.05	–0.53	0.20
24.9–27.9°*C*	0.92	0.46	0.19
≤ 25°*C*	*Reference category*		
Housing: Year structure (house) built in 2005 or later	1.83	0.74	0.20
Percent developed—medium intensity area	1.20	0.58	0.12

All covariates were in a continuous format with the exception of daytime land surface temperature, which was categorized.

**Table 5:** Model statistics for Bayesian spatio–temporal covariate models evaluating county–level RMSF prevalence in four central Midwestern states (Kansas, Missouri, Arkansas, Oklahoma), United States of America.

Covariate	*Model– 3*	*Model– 4*	*Model– 5*
*Estimate (95% Bayes Cr*. *I)*			
*β*_1_	0.31 (0.16, 0.41)	0.32 (0.16, 0.39)	0.31 (0.11, 0.41)
*β*_2_	–0.12 (–0.08, –0.13)	–0.13 (–0.09, –0.13)	–0.13 (–0.07, –0.12)
*β*_3_	0.13 (0.06, 0.15)	0.13 (0.07, 0.12)	0.14 (0.06, 0.14)
*β*_4_	0.74 (0.02, 0.91)	0.76 (0.02, 0.90)	–
*β*_5_	0.58 (0.05, 0.12)	–	–

*β*_1_ = poverty–status, *β*_2_ = daytime LST (≥ 35°*C*), *β*_3_ = relative humidity, *β*_4_ = number of houses built in 2005 or before, *β*_5_ = percent developed—medium intensity area.

**Table 6:** Model fit and comparison criteria.

Model	*$\bar{D}$*	*p*_*D*_	*DIC*	*LS*
*Partial spatio–temporal model*				
*Model*– 1: Non–parametric terms (*γ*_*j*_, *ψ*_*ij*_)	4654.21	358.31	5012.52	0.31, 0.57
*Model*– 2: Parametric terms (*β*_*j*_, *ψ*_*ij*_)	4012.34	302.11	4314.45	0.28, 0.51
*Covariate model*				
*Model*– 3: *Model*– 2 + *β*_1_,…, *β*_5_.	4723.59	402.61	5126.20	0.32, 0.59
*Model*– 4: *Model*– 2 + *β*_1_,…, *β*_4_.	3951.27	247.64	4198.91	0.25, 0.48
*Model*– 5: *Model*– 2 + *β*_1_,…, *β*_3_.	3429.73	236.18	3665.91	0.21, 0.43

$\bar{D}$ is the expected deviance, *p*_*D*_ is the deviance derived from the expected values of parameters, *DIC* is the deviance information criterion, and *LS* is the logarithmic score.

*β*_1_ = poverty–status, *β*_2_ = daytime LST, *β*_3_ = relative humidity, *β*_4_ = number of houses built in 2005 or before, *β*_5_ = percent developed—medium intensity area. The removal of *β*_2_, then *β*_1_ one at a time resulted in model *DIC* values of 3984.24 and 3746.65, respectively, and were therefore retained in the Bayesian covariate model.

The posterior Bayes estimates of the final covariate model indicated that all variables except the ‘number of houses built in 2005 or before’, and ‘percent developed—medium intensity area’ were significantly positively related to RMSF levels in the four states. Based on the magnitude of posterior median estimates, we observe that the socio–economic variable ‘poverty–level’ was most important, followed by almost identical influence of ‘average relative humidity’ and ‘average daytime land surface temperature’(≥ 35°*C*).

**Fig 2:**
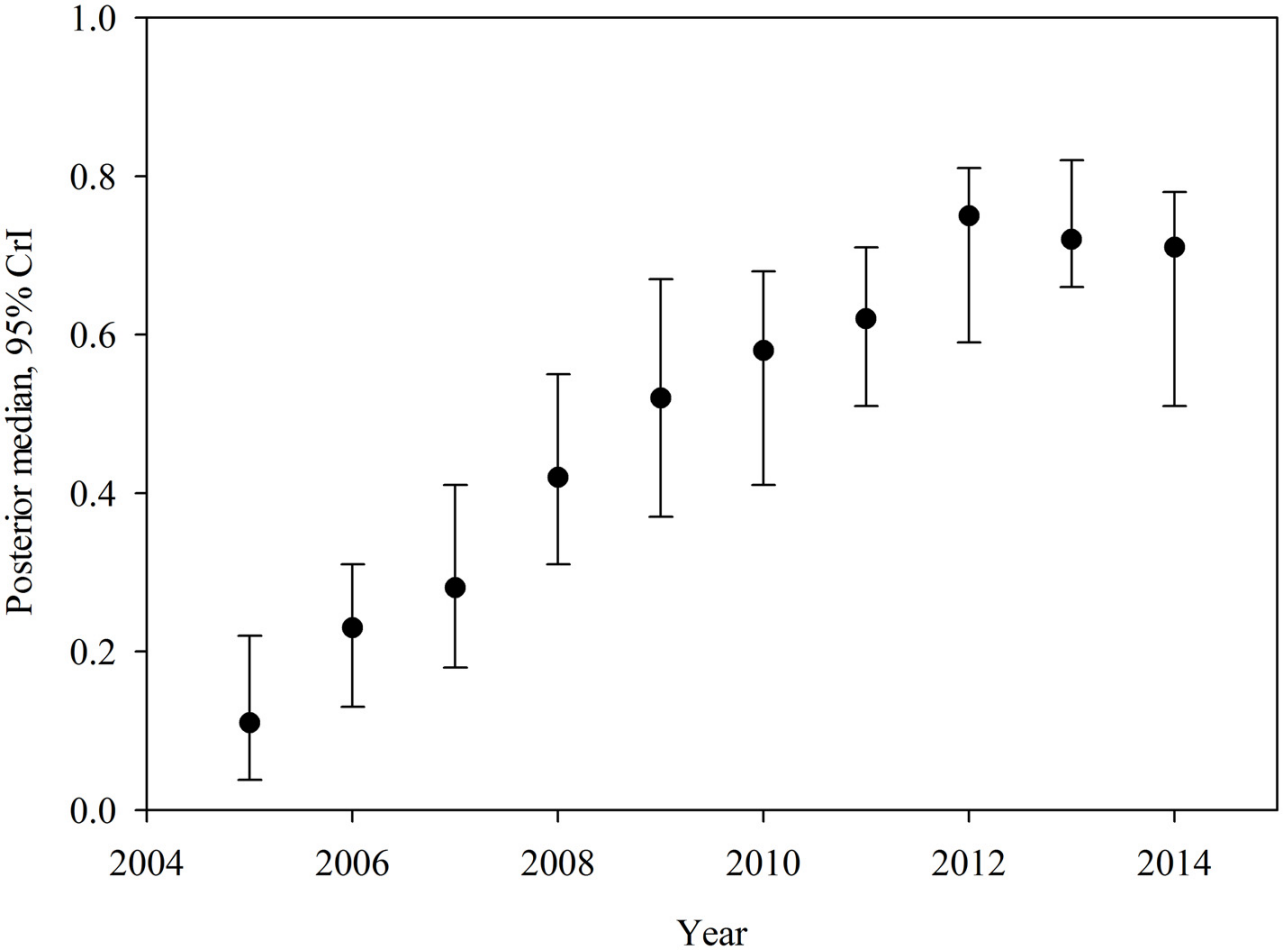
The posterior median and 95% CrI for the overall time trend in the covariate model.

**Fig 3:**
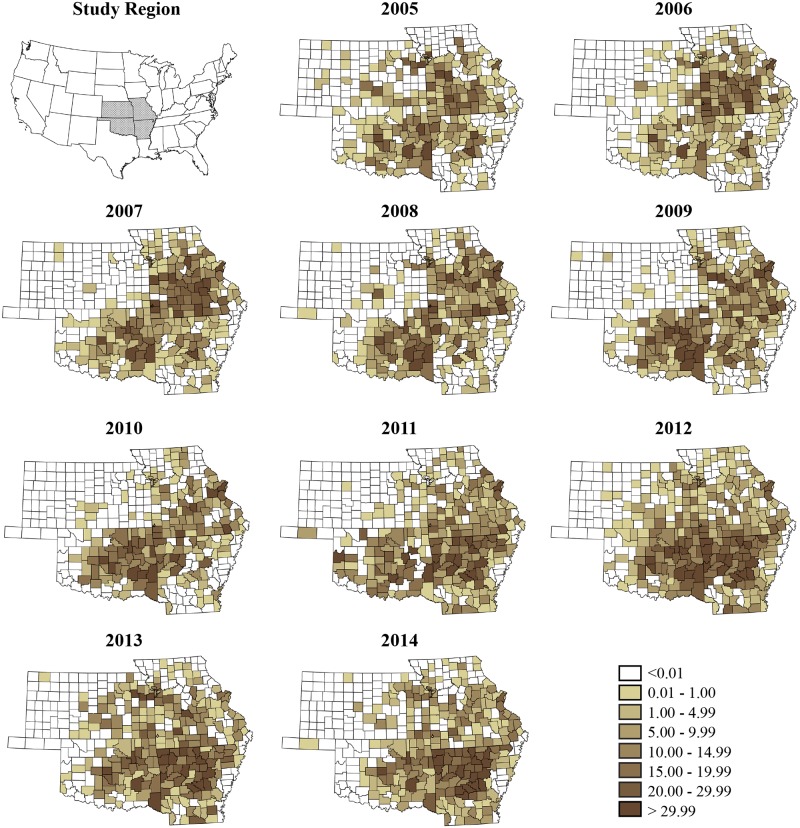
County–level crude rate estimates of Rocky Mountain spotted fever prevalence reported to the state health departments for the study period, 2005–2014.

**Fig 4:**
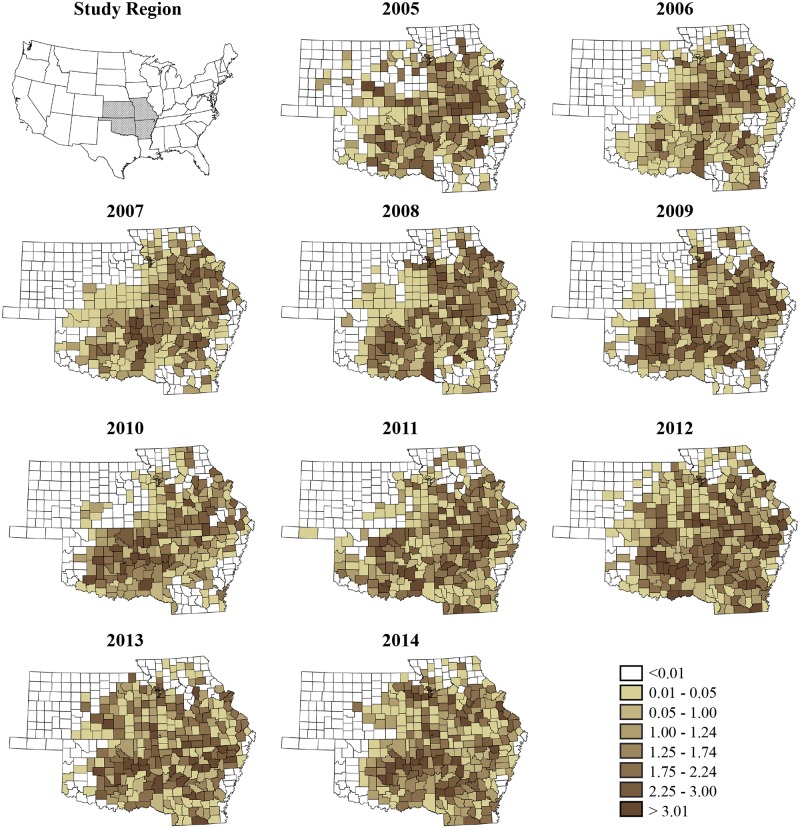
County–specific Bayesian smoothed estimates (posterior median) of Rocky Mountain spotted fever prevalence for the study period between years 2005–2014.

**Fig 5:**
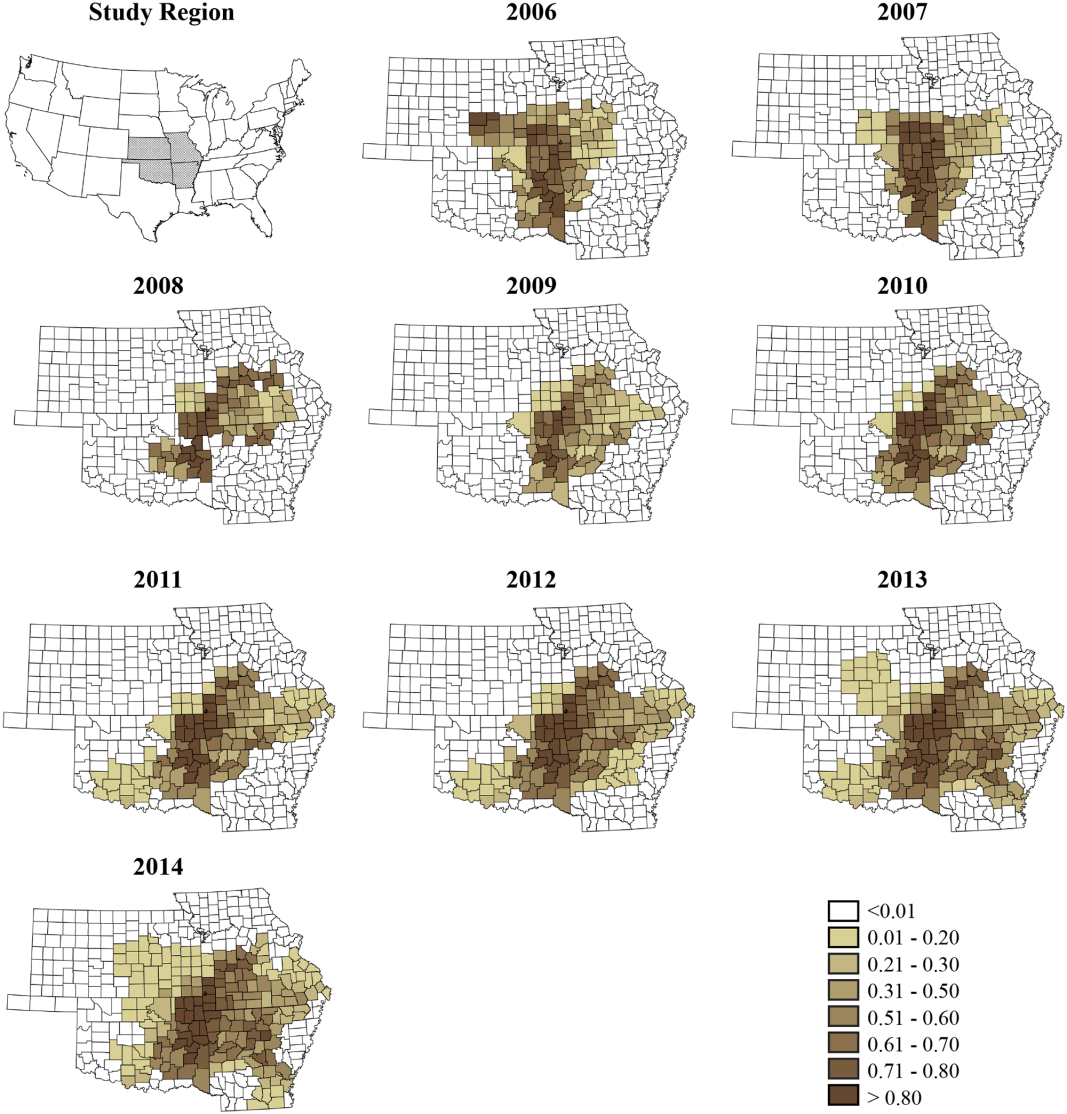
Posterior median of county–specific differential trends. Counties with values closer to 0 indicate a higher risk for RMSF.

The posterior median and uncertainty levels (95% CrI) of overall time trend, *β*_*j*_ is depicted in Fig 2, showing a clearly increasing trend for the region with a slight decrease in the later part of the study. Plots of annual crude rate estimates for RMSF prevalence for individual years over the study period is depicted in Fig 3, while Fig 4 illustrates maps of Bayes smoothed annual RMSF estimated relative risks for the study period after adjusting for the random terms and covariate effects. The estimates are based on the Bayesian geostatistical covariate model with socio–economic and climatic predictors and correspond to the median of the posterior predictive distribution. The counties of high risk were identified by mapping county–specific differential trends (posterior median of *Ψ*_*ij*_) with values greater than 0 (Fig 5) after accounting for covariate effects in the final model. Higher values close to zero indicate counties with a high probability of difference from the overall trend.

## 4. Discussion

The smoothed risk maps of relative risk produced in this study show all areas in the region that are affected by RMSF year after year, and are superior to maps of crude rate estimates, largely owing to the ability of Bayesian models to efficiently borrow information from neighboring areal units, and in tackling the low–number problem commonly associated with epidemiological data in some areal units versus others. A primary concern in this study however was to identify the presence of any overall trend for the region, and to isolate high risk counties where disease trend could be increasing. We therefore specified appropriate random effect terms in a Bayesian framework to identify an overall time trend, and any space–time interaction effect, followed by the addition of different covariates that would explain additional variability in the data due to space–time effect. Although a descriptive plot of the reported numbers of cases to the health departments (see Fig 1) show a declining trend for Missouri, and a more or less stable trend for Kansas, this study has identified a steadily increasing overall time trend for RMSF for all four contiguous states combined during the study period, indicating that RMSF has in fact increased year by year in the overall region, which are not visible when simply the crude numbers are plotted. Maps of counties with positive differential trends (*Ψ*_*ij*_ > 0) represent higher risk for RMSF compared to other counties in the region, and can be seen to have increased in area over the years and expanded outwards from a central focus in 2006 bordering all four states towards all the directions in subsequent years. These findings have implications for prevention and management strategies of RMSF.

The notable abrupt increase in the number of cases during the 2011–2012 period in Arkansas and Oklahoma could be attributed to a number of factors, including changes to reporting practices employed by the state health departments in their respective states that may have temporarily increased interest in case reporting, and as well as changes to diagnostic methods adopted by laboratories during this period, which may have improved the accuracy levels by which different tick–borne diseases are identified. It is evident through the state health departments of Arkansas and Oklahoma that they made a strong push for improving submission rates for tick-borne disease cases through awareness and education campaigns during the 2010–2011 period. Also, increasingly more diagnostic laboratories in the region are adopting molecular methods based on *in vitro* amplification procedures for rapidly detecting multiple tick-borne diseases, reasons that could have played a role in the noticed surge. The presence of environmental or climatic linkages behind this surge is also conceivable, but they could not be adequately evaluated in the present study due to data limitations. However, such evaluation of any associations, particularly with climatic patterns is worthy of further consideration. The winter and spring months of the year 2012 was unusually warm and humid in the Midwestern U.S [42], [43]. Even though a prolonged low pressure system over the Midwest lead to a drought in the summer of 2012, conditions earlier in the tick season may have favored a higher population growth and the proliferation of pathogens.

Poverty status of individuals in the four states was a strong predictor of RMSF in this study. In an earlier study we identified poverty status to be a significant predictor of another tick-borne disease, human monocytic ehrlichiosis in the same region as well [28]. Such associations of tick-borne diseases with socio–economic conditions are rarely evaluated in North America; however, lower socio–economic conditions were stronger predictors of tick-borne encephalitis in Europe compared to climatic and environmental predictors [23], [44]. This recurring association is perhaps indicative of higher exposure to tick pathogens for low–income individuals, who may work outdoors, and lower literacy levels and less awareness towards preventing tick-borne illnesses could be contributing factors.

There is notable interest in understanding the implications of ongoing climate–change on incidences of vector–borne diseases worldwide [10], [14], [45]. Climatic conditions affect arthropod phenology, population dynamics and their distribution over space, and the recent changes noted in shifting spatial range of some tick vectors [46], extended activity period due to relatively warmer conditions during winter [47], and changes to questing behavior all have been suspected to be influenced by climate–change [48], [49]. Whilst a strong emphasis is placed on researching the effect of climate change on disease–spreading arthropod vectors, the detection and then ascribing climate–change to increased disease incidences is however problematic since there are many confounding players involved in such systems. Even though ongoing climate change is conceivably an influential driver in vector–borne disease systems, its relative importance over social, economic and demographic factors is debated [23]. This study further shows that a mixture of factors are important in the spatio–temporal patterns or the eco-epidemiology of tick–borne diseases, including such factors as poverty status and they need to be accounted for in modeling climate–change implications on vector–borne diseases.

The two climatic factors associated with RMSF prevalence in this study, relative humidity and daytime land surface temperature are important climate–change indices, and are intensely monitored by climate–change researchers [50]. Their identification in the present study as important drivers of RMSF at a county–scale is therefore significant. Optimal humidity conditions are vital for tick survival and it is an important delimiter to their spatial distribution [51], and relative humidity can often be seen associated with the survival and abundance of ticks in the literature, with higher humidity conditions often favoring the long–term survival of some ticks species’ life stages through dry seasons [48] among other reasons. The higher daytime land surface temperature identified in this study is likely an indication of the average temperature conditions in some counties that are unfavorable for *Dermacentor* ticks versus others. Potential temperature effects on ticks and tick–pathogen interactions have been noted [52], and studies aiming to understand any physiological effects on *Dermacentor* ticks and *R*. *rickettsii* interaction in their host are worthy of consideration.

Some limitations of the study need to be mentioned. Even though *R*. *rickettsii* is identified as the causative agent for RMSF, multiple *Rickettsia* species are vectored by ticks—many known to infect humans, which lead to similar clinical symptoms to that of RMSF [53]. These other *Rickettsia* spp. also cross-react in diagnostic tests that are currently performed for confirming RMSF status, leading to poor test specificity [54]. The extent to which cases were wrongly identified as RMSF in the present dataset cannot be reliably verified, although a majority of the cases are likely to be *R*. *rickettsii* causing RMSF. We expect that the use of PCR–based diagnostic methods versus the current serology–based diagnosis will alleviate the misdiagnosis in the future and a clearer picture of the space–time dynamics of RMSF will become evident.

## 5. Conclusions

Results of this study show that Rocky Mountain spotted fever incidence in the central Midwestern states of Kansas, Missouri, Oklahoma and Arkansas have increased over the past decade, with high risk counties for this disease lying in the central and southern portions of the region. At the scale of a county, the spatial and spatio-temporal covariates of poverty level and average relative humidity are positively influential for incidence levels, while average day time land surface temperatures above 35°C is a limiting factor. Future epidemiological studies on this disease and other tick-borne diseases will benefit by considering socio-economic status of individuals, particularly poverty status. The identification of climate–change indices as important drivers of RMSF in this study is significant in the context of climate–change impacts on infectious diseases.
